# Data on polymorphisms in CYP2A6 associated to risk and predispose to smoking related variables

**DOI:** 10.1016/j.dib.2017.09.013

**Published:** 2017-09-13

**Authors:** Luis A. López-Flores, Gloria Pérez-Rubio, Alejandra Ramírez-Venegas, Enrique Ambrocio-Ortiz, Raúl H. Sansores, Ramcés Falfán-Valencia

**Affiliations:** a Laboratorio HLA, Instituto Nacional de Enfermedades Respiratorias Ismael Cosío Villegas, México City, Mexico; b Departamento de Investigación en Tabaquismo y EPOC, Instituto Nacional de Enfermedades Respiratorias Ismael Cosío Villegas, México City, Mexico; c Centro Respiratorio de México, Mexico

**Keywords:** CYP2A6, Smoking, Nicotine addiction

## Abstract

This article contains data on the single nucleotide polymorphisms (SNPs) rs1137115, rs1801272 and rs28399433 rs4105144 in *CYP2A6* associated to smoking related variables in Mexican Mestizo smokers (Pérez-Rubio et al., 2017) [1]. These SNPs were selected due to previous associations with other populations. Mexican Mestizo smokers were classified according their smoking pattern. A genetic association test was performed.

**Specifications Table**

**Table:** 

Subject area	Genomic medicine
More specific subject area	Genetic epidemiology
Type of data	Table and figure
How data was acquired	Smoking pattern survey, allelic discrimination assay by real-time PCR (Applied Biosystems, Foster City, CA, USA).
Data format	Analyzed (Fig. 1, Fig. 2, Fig. 3 and Table 1)
Experimental factors	Peripheral blood sample, DNA extraction by BDtract DNA isolation kit (Maxim Biotech, Inc. San Francisco, California, USA).
Experimental features	Genotyping was performed using 3 μL of DNA at 15 ng/μL concentration and TaqMan probes (Applied Biosystems Foster City CA, USA). In each template, we included 3 non-template controls, and 1% of the samples were genotyped in duplicate as an allele assignment control.
Data source location	Instituto Nacional de Enfermedades Respiratorias Ismael Cosío Villegas (INER) at México City
Data accessibility	Accessible from this article; DNA sample and raw data are available for further analyses in collaborative studies.

**Value of the data**

• Genetic association studies in Latin American populations as Mexican mestizos are scarce and show distinct values due to the admixture in the genetic structure.
• Mexican mestizo smokers exhibit a different smoke pattern compared with other populations.
• There are few data about genetic risk for smoking behavior associated with *CYP2A6* in Mexican mestizo population.
• Mexican mestizo smokers who carry some risk alleles in *CYP2A6* could predispose to smoking behavior variables.

## 1 Data

Single nucleotide polymorphisms (SNPs) rs1137115, rs1801272 and rs28399433 rs4105144 in *CYP2A6* associated to smoking related variables in Mexican Mestizo smokers [1].

**Fig. 1:**
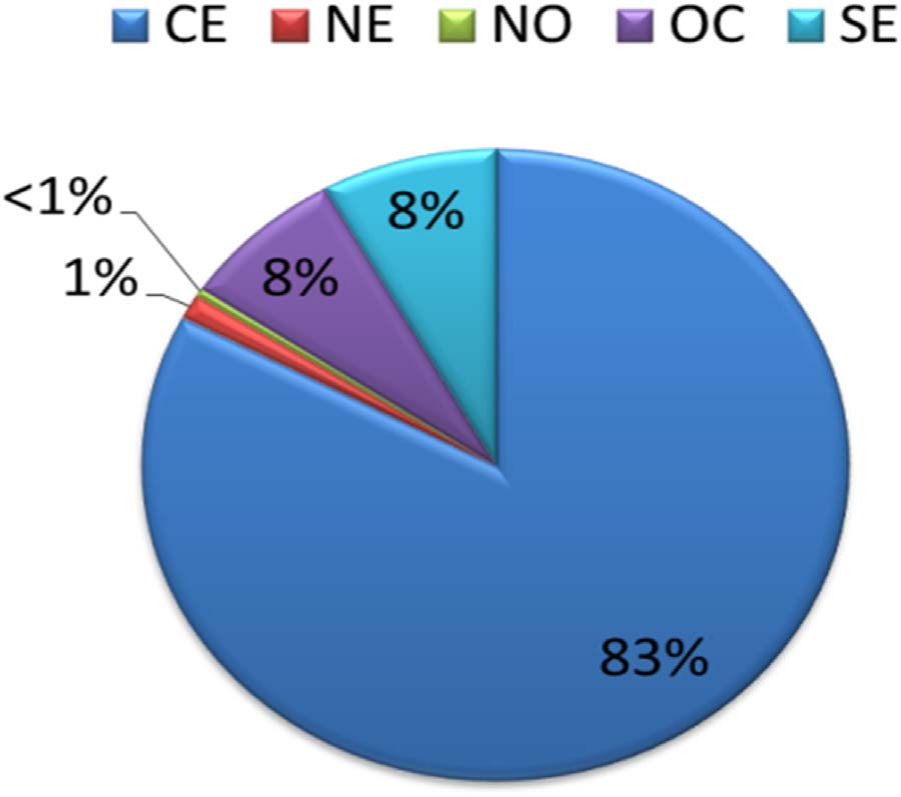
Classification of subjects according to their birthplace geographic region.

Mexican Mestizo subjects were classified into five groups according to their birthplace geographic region in: Northwest (NW; Baja California, Baja California Sur, Chihuahua, Sinaloa and Sonora), Northeast (NE; Coahuila, Durango, Nuevo León, San Luis Potosí and Tamaulipas), West (WE; Aguascalientes, Colima, Guanajuato, Jalisco, Michoacán, Nayarit, Querétaro and Zacatecas), Central (CE; Mexico city, Mexico state, Guerrero, Hidalgo, Morelos, Puebla and Tlaxcala) and Southeast (SE; Campeche, Chiapas, Oaxaca, Quintana Roo, Tabasco, Veracruz and Yucatán). Most of the participants were from CE (83%), followed by WE and SE (8% each), and NE and NW had a minor proportion (<1%) (Fig. 1).

**Table 1:** Codominant model analysis for genotypes and alleles of SNPs analyzed in *CYP2A6*.

SNP Genotype or allele	HS (*n*=351)		LS (*n*=349)		NS (*n*=394)	
	*n*	GF/AF (%)	*n*	GF/AF (%)	*n*	GF/AF (%)
rs1137115						
GG	234	66.67	240	68.77	296	75.12
GA	107	30.48	103	29.51	89	22.58
AA	10	2.85	6	1.72	9	2.31
G	575	81.90	583	83.52	681	86.42
A	127	18.10	115	16.48	107	13.58
						
rs4105144						
CC	227	64.67	232	66.48	284	72.08
CT	112	31.90	103	29.51	99	25.12
TT	12	3.41	14	4.01	11	2.79
C	566	80.62	567	81.23	667	84.64
T	136	19.37	131	18.76	121	15.36
						
rs1801272						
TT	341	97.15	337	96.56	389	98.73
TA	10	2.85	11	3.14	5	1.27
AA	0	0	1	0.28	0	0
T	692	98.57	685	98.14	783	99.36
A	10	1.42	13	1.86	5	0.63
						
rs28399433						
TT	254	72.36	242	69.34	277	70.30
TG	88	25.07	94	26.93	110	27.91
GG	9	2.56	13	3.72	7	1.77
T	596	84.9	578	82.80	664	84.26
G	106	15.09	120	17.19	124	15.73

HS, Heavy smokers; LS, Light smokers; NS, Never-smokers; GF, Genotypic frequency; AF, Allelic frequency.

## 2 Experimental design, materials and methods

### 2.1 Subjects

We selected subjects with ≥40 years old, men and women. To determine Mexican Mestizo ancestry, subjects were asked about their parents and grandparents ancestry and not belong to an indigenous group.

Smokers recruited from the Smoking Cessation Support Clinic of the Department of Investigation in Tobacco Consumption and COPD at the Instituto Nacional de Enfermedades Respiratorias Ismael Cosío Villegas (INER) in Mexico City, with the following criteria: had 10≥years smoking were classified according their smoking pattern in cigarette smoking per day (cpd) into light smokers (LS≤10 cpd) and heavy smokers (HS≥20 cpd), these subjects has been recruited previously in our research group [2], [3]. A reference group of never-smokers healthy volunteers was selected with the same demographic characteristics.

### 2.2 Smoking pattern variables

A survey with smoking related variables were assed to daily smokers. They were asked about their age at onset smoking, cigarette smoking per day (cpd) and years of smoking.

### 2.3 DNA extraction

A 15 mL peripheral blood sample in tubes with EDTA was obtained from each participant through venipuncture. DNA extraction was performed using BDtract DNA isolation kit (Maxim Biotech, Inc. San Francisco, California, USA) and later was quantified with a NanoDrop 2000 (Thermo Scientific, DE, USA). DNA samples with a concentration >100 ng/mL and purity with a 260/280 relation ≥2.

### 2.4 SNP selection

We searched publications from 2010 to 2015 on genetic association studies performed in Caucasian, Asian and African populations. We identified rs1137115, rs1801272 and rs28399433 in *CYP2A6* and rs4105144 near the gene.

### 2.5 Genotyping

**Fig. 2:**
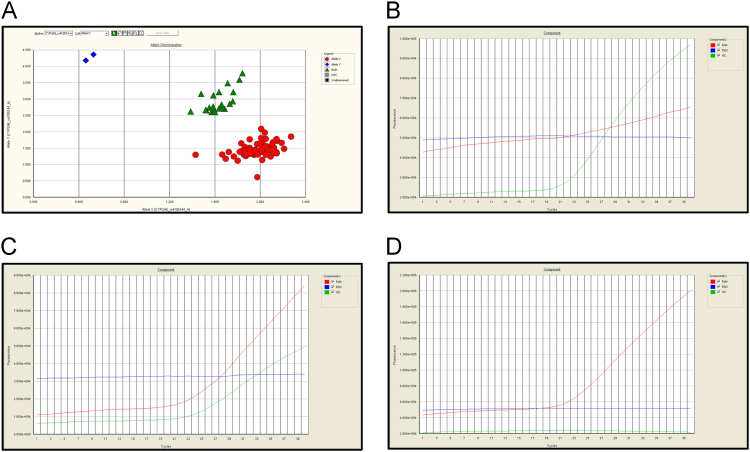
Real-time PCR discrimination assay by TaqMan® probes. (A) Allelic discrimination plot. Subjects were designated in three groups according to the dye detected by the system: (B) Allele X or genotype by dyes VIC™/ VIC™. (C) Both alleles (X and Y) or genotype by dyes VIC™/FAM™. (D) Allele Y or genotype by dyes FAM™/ FAM™.

Genotyping was performed using a real-time PCR (7300 Real-Time PCR system, Applied Biosystems, Foster City, CA, USA) based allelic discrimination assay using 3 μL of DNA at 15 ng/μL concentration and TaqMan probes (Applied Biosystems Foster City CA, USA). In each template, we included 3 non-template controls, and 1% of the samples were genotyped in duplicate as an allele assignment control. Sequence Detection Software (SDS v. 1.4, Applied Biosystems). VIC™ and FAM™ dyes were used for alleles A and B, respectively (Fig. 2).

**Fig. 3:**
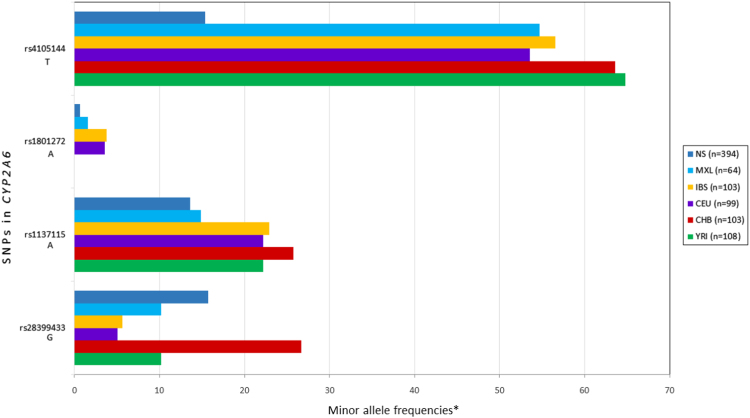
Minor allele frequencies for *CYP2A6* SNPs in NS compared with 1000 Genomes Project populations. NS, Never-smokers Mexican Mestizo; IBS, Iberian Population in Spain; MXL, Mexican Ancestry in Los Angeles CA; CEU, Utah residents with Northern and Western European ancestry; CHB, Han Chinese in Beijing China; YRI, Yoruba in Ibadan, Nigeria. * in NS group.

### 2.6 Statistical analyses

To describe the study population, we used the statistical software SPSS v.20.0 (IBM, New York, USA) in which we calculated the mean and standard deviation of each continuous quantitative variable and the percentage for the genre. All SNPs genotyped were evaluated in the control group (NS) using the Hardy-Weinberg test. Haplotype analysis was performed using Haploview version 4.2.

### 2.7 Genetic association

Genetic association was tested in different models: full genotype, dominant, recessive and by allele using Epidat version 3. To identify SNPs associated with increased nicotine addiction, we compared HS *vs.* LS, and to associate SNPs with cigarette consumption, we compared HS *vs.* NS and LS *vs*. NS (Table 1, Fig. 3).

## Ethical approval

The protocol was approved by INER science and research bioethics and biosecurity committees (protocol number B15-16).
